# Multivariate and repeated measures (MRM): A new toolbox for dependent and multimodal group-level neuroimaging data

**DOI:** 10.1016/j.neuroimage.2016.02.053

**Published:** 2016-05-15

**Authors:** Martyn McFarquhar, Shane McKie, Richard Emsley, John Suckling, Rebecca Elliott, Stephen Williams

**Affiliations:** a Neuroscience & Psychiatry Unit, Stopford Building, The University of Manchester, Oxford Road, Manchester M13 9PL, UK; b Centre for Biostatistics, Jean McFarlane Building, The University of Manchester, Oxford Road, Manchester M13 9PL, UK; c Brain Mapping Unit, Herchel Smith Building for Brain and Mind Sciences, University of Cambridge, Robinson Way, Cambridge CB2 0SZ, UK; d Imaging Sciences, Stopford Building, The University of Manchester, Oxford Road, Manchester M13 9PL, UK

**Keywords:** Multivariate GLM, Permutation, Multimodal, Repeated measures, Discriminant functions

## Abstract

Repeated measurements and multimodal data are common in neuroimaging research. Despite this, conventional approaches to group level analysis ignore these repeated measurements in favour of multiple between-subject models using contrasts of interest. This approach has a number of drawbacks as certain designs and comparisons of interest are either not possible or complex to implement. Unfortunately, even when attempting to analyse group level data within a repeated-measures framework, the methods implemented in popular software packages make potentially unrealistic assumptions about the covariance structure across the brain. In this paper, we describe how this issue can be addressed in a simple and efficient manner using the multivariate form of the familiar general linear model (GLM), as implemented in a new MATLAB toolbox. This multivariate framework is discussed, paying particular attention to methods of inference by permutation. Comparisons with existing approaches and software packages for dependent group-level neuroimaging data are made. We also demonstrate how this method is easily adapted for dependency at the group level when multiple modalities of imaging are collected from the same individuals. Follow-up of these multimodal models using linear discriminant functions (LDA) is also discussed, with applications to future studies wishing to integrate multiple scanning techniques into investigating populations of interest.

## Introduction

Group-level repeated measurements are commonplace in neuroimaging research, from neurocognitive paradigms with multiple activation conditions to longitudinal intervention studies. Despite this, conventional summary statistic approaches to modelling these data ignore the repeated measurements in favour of the construction of contrasts at the subject level. These contrasts are then explored using multiple group-level linear models. Though this approach is advantageous due to its simplicity, when the design contains more than two repeated measurements many of the typical ANOVA tests used to investigate the repeated measures and their interactions are either overly complex to implement or simply not possible. Furthermore, for approaches such as the *p*-block method of analysing pharmacological challenge fMRI data (phMRI; e.g. McKie et al., 2011), the use of contrasts at the individual-level is not a useful method and repeated-measurement models become a necessity. Despite this, the approaches currently implemented in two of the most popular fMRI analysis packages, FSL (http://fsl.fmrib.ox.ac.uk/fsl/) and SPM (http://www.fil.ion.ucl.ac.uk/spm/), are not able to easily account for dependent group-level neuroimaging data. FSL FEAT must assume sphericity at every voxel so that *F*-tests follow an exact *F*-distribution (Huynh and Feldt, 1970). Cases where the sphericity condition is not met can lead to a poorer control of the type I error rate due to overly liberal *F*-statistics (Box, 1954, Kogan, 1948). SPM, on the other hand, has a method for correcting departures from sphericity (Glaser and Friston, 2007). However, the estimated structure used in this correction is assumed to be the same for every voxel. In both cases, these assumptions may not always be valid for complex dependent data.

Further to the issues of dependent group-level analyses, it is also commonplace to collect multiple imaging sequences from the same subjects during the same scanning session (e.g. functional, T1 structural, arterial spin labelling). In some cases, there may even be different modalities of imaging collected from the same individuals (e.g. MR and PET). Analysing these different sequences/modalities is similar to repeated-measures designs due to the assumed correlation between measurements taken from the same individual. The biggest difference with repeated-measurement models is simply that the data are not guaranteed to be commensurate as they are generally not measured on the same scale. Although questions of interest often focus on the sequences and modalities individually, pooling the information provided by different imaging techniques may be advantageous in exploring how a combination of measurements may provide information on group differences above and beyond the information they provide individually. To achieve this, methods that accommodate both the assumed correlation and the differing scales of the measurements are needed.

In this paper, we will demonstrate how both the issues of repeated-measures and multimodal^1^ group models can be addressed using the multivariate form of the familiar univariate general linear model (GLM). We introduce a MATLAB toolbox for fitting these models called Multivariate and Repeated Measures (MRM), comparing results from real neuroimaging datasets between this approach and other implementations of repeated-measures modelling of neuroimaging data. We also highlight the ability of this approach to integrating multimodal group-level imaging datasets. In addition, we discuss facilities in the MRM software to perform descriptive linear discriminant analysis (*d*LDA) to investigate how information from different modalities and sequences can be combined to maximally separate groups of interest. We also discuss the use of permutation-based approaches to *p*-value calculation, and multiple comparison corrections at both the voxel and cluster level, highlighting the utility of these methods when applied to the multivariate GLM.

## Theory

The theory behind the multivariate extension of the univariate GLM is well documented (Christensen, 2001, Davis, 2002, Rencher and Christensen, 2012), and has recently been advocated for use in neuroimaging by Chen et al. (2014). Here we present a brief overview for completeness, emphasising how this approach is naturally adapted for repeated-measures/longitudinal models as well as multimodal integration. We also present the theory behind *d*LDA as an extension of the multivariate framework for understanding the contribution of multimodal imaging data to the separation of groups of interest.

### The multivariate GLM

The multivariate form of the univariate GLM is expressed as

(1) Y=XB+E

where **Y** is an *n* × *t* matrix of observations, **X** is the *n* × *k* design matrix, **B** is the *k* × *t* matrix of model parameters, and **E** is the *n* × *t* matrix of errors. This can be written in matrix form as

(2) $$\left(\begin{array}{c} Y_{11}\\ \vdots \\ Y_{n1} \end{array}\;\begin{array}{c} \cdots \\ \cdot \\ \ldots \end{array}\;\begin{array}{c} Y_{1t}\\ \vdots \\ Y_{\mathrm{nt}} \end{array}\right)=\left(\begin{array}{ccc} \begin{array}{c} x_{11}\\ \vdots \\ x_{n1} \end{array} & \begin{array}{c} \ldots \\ \cdot \\ \ldots \end{array} & \begin{array}{c} x_{1k}\\ \vdots \\ x_{\mathrm{nk}} \end{array} \end{array}\right)\;\left(\begin{array}{ccc} \beta_{11} & \ldots & \beta_{1t}\\ \vdots & \cdot & \vdots \\ \beta_{k1} & \ldots & \beta_{\mathrm{kt}} \end{array}\right)+\left(\begin{array}{ccc} \begin{array}{c} \epsilon_{11}\\ \vdots \\ \epsilon_{n1} \end{array} & \begin{array}{c} \ldots \\ \cdot \\ \ldots \end{array} & \begin{array}{c} \epsilon_{1t}\\ \vdots \\ \epsilon_{\mathrm{nt}} \end{array} \end{array}\right)$$

where *n* can be taken as the number of subjects, *t* as the number of *dependent variables*, here referred to as the repeated measurements or modalities, and *k* as the number of *independent variables*, here referred to as the predictors. Traditionally, it is assumed that $Y_{i}\sim N\;\left(X_{i}B,\Sigma \right)$ so that each *i*th row of **Y** is considered drawn from a multivariate normal distribution with a mean vector given by ***X***_*i*_**B**, and an unstructured covariance matrix **Σ**. As with the univariate case, these assumptions can more usefully be expressed using the errors so that

(3) $$\mathrm{Vec}\left(E\right)\sim N\left(0,I_{n}⊗\Sigma \right)$$

where the Vec operator is used to re-express a matrix as a vector by stacking the transposed rows (Christensen, 2011, Rencher and Christensen, 2012). Here **0** is a vector of zeros, ***I***_*n*_ is the *n* × *n* identity matrix, and ⊗ denotes the Kronecker product.

Estimation of **B** is usually performed using ordinary least squares,

(4) $$\hat{B}={\left(X'X\right)}^{-1}X'Y$$

identical to performing *t* univariate estimates using the columns of **Y**. Here, the most salient difference with univariate approaches is evident as we no longer have a *vector* of estimated parameters but a *matrix*, with one column for each of the *t* dependent variables and one row for each of the *k* predictors in **X**. Calculation of the multivariate residuals follows using $\hat{E}=Y-X\hat{B}$ so that an unbiased estimate of **Σ** can be made using

(5) Σ^=1n-kE^'E^

(Davis, 2002, Rencher and Christensen, 2012). Here we see that the covariance structure of the model is both unconstrained and very simple to estimate. When applied to imaging data the residual matrix $\hat{E}$ is estimated on a per-voxel basis and thus it is trivial to estimate a unique covariance structure for every voxel. This is a distinct advantage of mass multivariate approaches to dependent neuroimaging data. However, it should be clear from Eq. (3) that in this framework the covariance structure is assumed identical across groups. We shall return to this issue later.

The multivariate framework allows for the modelling of both repeated-measures and multimodal group-level imaging data. In both instances, each row of **Y** represents measurements from a single subject (for a particular voxel), with the columns of **Y** representing the multiple observations for that subject. Whether modelling repeated measurements or multiple modalities, there is an assumed degree of correlation between the columns of **Y**. This correlation is expressed using the estimated variance–covariance matrix $\hat{\Sigma }$, as indicated above. The utility of mixed-effects approaches for dependent data is in part due to their flexibility in specifying a variety of covariance structures (Mcculloch et al., 2008, Searle et al., 1992), whereas the assumption of a spherical covariance structure is one of the main reasons the traditional repeated-measures ANOVA approach is typically avoided (Davis, 2002). In the multivariate approach, an unconstrained covariance structure at every voxel provides the opportunity for inference without making any assumptions on the form that the covariance may take across the brain. As such, we argue that this is the safest approach without the computational burden of estimating variance components using iterative maximum-likelihood at every voxel (Guillaume et al., 2014). Notably, such a structure can also be fit uniquely at each voxel using marginal models, where the covariance structure is treated as a nuisance factor, allowing simplification of the mixed-effects scheme where both fixed and random effects must be specified directly (Guillaume et al., 2014, Li et al., 2013, Skup et al., 2012).

Extension of the multivariate GLM to accommodate continuous covariates is identical to the univariate domain and simply involves adding the, usually mean-centred (Poldrack et al., 2011), covariate *w*_*i*_ as another column in the design matrix **X**. The parameters associated with *w*_*i*_ are therefore slopes of the relationship between *w*_*i*_ and **Y** for each column of **Y**. If a grouping variable is used to split the covariate then a per-condition, or per-modality, slope is estimated for each group separately. Comparisons of changes in slope across groups are then easily specified. This scheme is more straightforward than integrating continuous covariates into traditional univariate approaches to repeated measurements, although it does not allow for the specification of time-varying covariates. With no groups and only continuous covariates the model becomes a multivariate regression (see Rencher and Christensen, 2012).

### Hypothesis testing

Hypothesis testing in the multivariate GLM is based on the contrast

(6) $$\mathrm{AB}C^{'}=0$$

Here, the univariate scheme is extended by combining standard hypotheses on the *rows* of **B**, coded by the matrix **A**, with hypotheses on the *columns* of **B**, coded by the matrix **C**. For multivariate ANOVA (MANOVA) models contrasts of main effects and interactions involve setting **C** = **I**_*t*_, the *t* × *t* identity matrix, as the dependent variables are not assumed to be commensurate. This is the scheme most suitable for multimodal neuroimaging applications. For repeated-measures models the variables are guaranteed to be commensurate and comparisons between the measurements are usually of interest. As such, **C** can take on a number of forms. Here the hypothesis testing approach can be conceptualised as combining hypotheses about the groups using **A**, and hypotheses about the repeated measures using **C**. As an example, and assuming a cell-means coded design matrix, an interaction between 2 groups with 3 repeated-measurements per-subject can easily be specified with $A=\left(\begin{array}{cc} 1\; & -1 \end{array}\right)$ and $C=\left(\begin{array}{ccc} 1 & -1 & \;0\\ 0 & \;1 & -1 \end{array}\right)$. This is simply a combination of a between-subject main effect and within-subject main effect. Setting $A=\left(\begin{array}{cc} 1/2 & 1/2 \end{array}\right)$ would provide the within-subject main effect alone, with $C=\left(\begin{array}{ccc} 1/3 & 1/3 & 1/3 \end{array}\right)$ providing the between-subject main effect alone. In each case the effects of no interest are simply averaged. This scheme is also particularly flexible as the standard univariate GLM analyses on the individual dependent variables can be recovered using e.g. $C=\left(\begin{array}{ccc} 1 & 0 & 0 \end{array}\right)$.

### Test statistics in the multivariate GLM

Whether a repeated-measures or MANOVA model, the calculation of test statistics from the multivariate GLM is identical. There is a choice of four standard test statistics that can be constructed based on the calculation of two *sums-of-squares and cross products* (SSCP) matrices. For any particular contrast, there is an SSCP matrix associated with the hypothesis.

(7) $$\mathrm{SSC}P_{H}=\left(A\hat{B}C'\right)'{\left[A{\left(X'X\right)}^{–1}A'\right]}^{–1}\left(A\hat{B}C'\right)$$

and an SSCP matrix associated with the error

(8) $${\mathrm{SSCP}}_{E}=C\left({\hat{E}}^{'}\hat{E}\right)C'$$

These matrices are generalisations of the numerator and denominator sums-of-squares from the univariate GLM hypothesis-testing framework (Green et al., 1999, Searle, 1987). For example, when **C** = **I**_*t*_ the main diagonal of **SSCP**_*H*_ contains the sums of squares for the hypothesis in **A** as applied to the estimated parameters for each dependent variable separately. When **C** ≠ **I**_*t*_ , these are the sums of squares for the linear combinations of parameters across the dependent variables, as given by the rows of **C**. For univariate cases, or when **YC**' reduces to univariate form, **SSCP**_*H*_ becomes the single sums of squares for the hypothesis. Similarly, when **C** = **I**_*t*_ , the **SSCP**_*E*_ matrix is simply an unscaled form of the estimated covariance matrix $\hat{\Sigma }$. When **C** ≠ **I**_*t*_ , the **SSCP**_*E*_ matrix is the appropriate linear combination of unscaled variances and covariances dictated by the form of **C**. When there is only one dependent variable, the **SSCP**_*E*_ returns to the univariate residual sums of squares, demonstrating that the univariate GLM is simply a special case of the multivariate framework.

Construction of a test statistic from this hypothesis-testing scheme can be done in a number of ways. Generally speaking, the different methods all rely on some linear combination of the *q* eigenvalues (*λ*_1_, … , *λ*_*q*_) of **SSCP**_*E*_^-1^**SSCP**_*H*_. The four standard tests statistics (attributable to Hotelling, 1951, Lawley, 1938, Pillai, 1955, Roy, 1945, Wilks, 1932) are

(9) Pillai'strace=traceSSCPH+SSCPE−1SSCPH=∑i=1qλi1+λiWilks'lambda=SSCPESSCPH+SSCPE=∏i=1qλi1+λiHotelling‐Lawleytrace=traceSSCPE−1SSCPH=∑i=1qλiRoy'slargestroot=λ∗1+λ∗

where *λ*^⁎^ is the largest eigenvalue of ***SSCP***_*E*_^-1^***SSCP***_*H*_. Approximations to an *F*-statistic and the corresponding degrees of freedom can be calculated for all these statistics, allowing the designation of an approximate *p*-value (see Christensen, 2001, Rencher and Christensen, 2012 for derivation). It should be noted, however, that the *F* approximation for Roy's largest root is an upper-bound on the true *F*. As such, it carries with it the greatest type I error risk and generally is only safe to interpret for those tests where the null hypothesis is not rejected (see Rencher and Christensen, 2012, p. 165). A further point for neuroimaging is that all hypothesis tests in the multivariate GLM framework are based on *F*-statistics, meaning that it is not possible to test directional (one-tailed) hypotheses. See Appendix A for discussion on the choice between these test statistics. Later, we present some comparisons between these tests within a neuroimaging setting.

### Descriptive linear discriminant analysis (dLDA)

When using MANOVA models, the calculation of a sufficiently large multivariate test statistic naturally leads to the question of the degree to which any of the dependent variables are contributing to the rejection of the null hypothesis. Although it is possible to simply follow up any significant multivariate tests with multiple univariate tests this is generally discouraged (Rencher and Christensen, 2012, Tabachnick and Fidell, 2007). An approach more closely tied to the calculation of the MANOVA test statistics known as LDA is more favourable. The use of LDA as a follow-up tool for MANOVA models is well documented (Huberty and Olejnik, 2006, Klecka, 1980, Rencher and Christensen, 2012), and can either take the form of *d*LDA or *predictive* LDA (Hastie et al., 2009, Rencher and Christensen, 2012). Here, we focus on *d*LDA as a tool for indicating the relative importance of each dependent variable to group separation.

The *d*LDA approach is a reversal of the MANOVA model, seeking those linear combinations of dependent variables that best separate the specified groups. Formally, the *d*LDA model is given as

(10) $$z_{\mathrm{ij}}=a_{1}y_{\mathrm{ij}1}+a_{2}y_{\mathrm{ij}2}+\cdots +a_{t}y_{\mathrm{ijt}}=a'Y_{\mathrm{ij}}$$

where ***Y***_*ij*_ is the column vector of responses for subject *j* (*j* = 1 … *n*_*i*_), from group *i* (*i* = 1 … *k*), measured on *t* dependent variables (*p* = 1 … *t*). As with the traditional MANOVA model, it is assumed that each ***Y***_*ij*_ is drawn from a multivariate normal distribution with a group-dependent mean vector and a common covariance matrix. The weights in vector **a** represent a discriminant function and are calculated so that the transform of the multivariate response in ***Y***_*ij*_ to the scalar *z*_*ij*_, the *discriminant score*, maximises the standardised group difference on *z*_*ij*_. For example, for two groups, **a** is estimated to maximise $\left({\overset{-}{z}}_{1}-{\overset{-}{z}}_{2}\;\right)/s_{z}$, where ${\overset{-}{z}}_{i}$ denotes a mean for group *i* and *s*_*z*_ denotes the pooled standard deviation. The absolute values of the weights in **a** are therefore of interest as they indicate the contribution of each dependent variable to maximising the difference between the groups. For multimodal neuroimaging data, this allows a quantification of the degree to which each modality is able to contribute to group separation at a particular voxel. See Appendix B for more details.

## Approaches to inference and multiple comparison correction

In basic voxel-by-voxel neuroimaging analyses, a key point of contention is the multiple comparison problem engendered by testing across a large number of voxels. Application of the multivariate GLM to neuroimaging data is no exception. In addition, there may be some concern that the test statistics are only approximately *F* distributed, and therefore only provide approximate *p*-values. In the MRM software, we make use of permutation testing as a method of improving this approximation (Finch and Davenport, 2009), and as a method of providing a family-wise error (FWE) analogue to standard Gaussian random field (GRF) theory approaches to multiple testing in neuroimaging (Worsley et al., 1996). Although GRF results exist for some multivariate test statistics (Cao and Worsley, 1999, Carbonell et al., 2011, Taylor and Worsley, 2008), the permutation approach provides much greater flexibility. Adopting a permutation approach allows us to relax the distributional assumptions about the outcome data as well as use non-standard statistics, irrespective of their tractability under the null. Although we currently restrict this to cluster size and cluster mass (Bullmore et al., 1999), the framework provides flexibility to use many other statistics in the future, so long as they meet the condition of *pivotality* (Winkler et al., 2014). Here cluster size is simply the number of voxels within a cluster, defined using some cluster-forming threshold and a cluster counting scheme. Cluster mass, on the other hand, allows one to make use of the voxel-level information in the image by summing the test-statistics within a cluster, an approach that appears more sensitive (Bullmore et al., 1999). Using the multivariate framework also allows for a relatively easy solution to the problem of permutations under dependence, foregoing the specification of exchangeable blocks of data as necessitated by univariate approaches (Winkler et al., 2014). See Appendix C for details on the implementation in MRM.

## Software

**Fig. 1:**
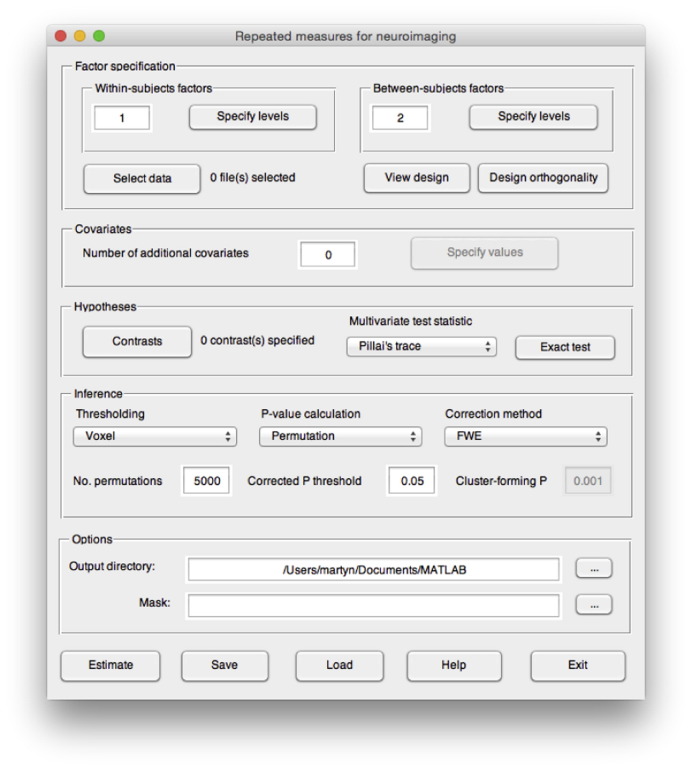
The main interface of the MRM software used to specify a repeated-measures group model.

The MRM software is a MATLAB-based toolbox designed for the specification of mass multivariate group models of neuroimaging data using the summary statistic approach. Fig. 1 shows the main window used for specifying a repeated-measurement model. Contrasts, following Eq. (6), are user specified in terms of the weights in matrices **A** and **C** for the general linear hypothesis test **ABC**^'^ = 0. There is also an auto-generation procedure for creating standard MANOVA and repeated-measures contrasts of main effects and interactions for arbitrary designs up to a 4-way interaction. Any number of continuous covariates measured at the between-subjects level are easily added to the design matrix, with automatic mean-centring conducted by default. This mean-centring can be switched off at the user's discretion. For the *d*LDA follow-up, all covariates are removed from the design prior to estimation. A number of options for inference are available including thresholding at both the voxel and cluster level, as well as using permutation methods to generate *p*-values that can be corrected using an FDR procedure, or used to provide a FWE correction. For permutation inference, the use of the randomise algorithm (detailed in Appendix C) allows permutation in the presence of nuisance covariates by orthogonalising the data with respect to the nuisance partition of the model. It is also possible to provide a mask in order to restrict inference to pre-defined regions of interest.

**Fig. 2:**
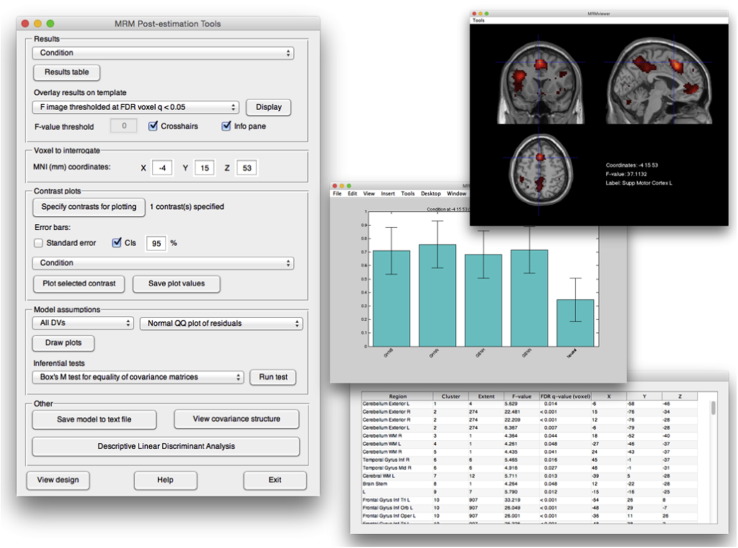
The MRM post-estimation tools.

After model estimation, the MRM post-estimation tools are available to explore results. These facilities are shown in Fig. 2 and include interactive assessment of thresholded maps, plots of linear combinations of the model parameters, and model assumption checking. The checking of parametric assumptions is rarely conducted in neuroimaging data analysis, an issue discussed by a number of authors (Poline and Brett, 2012, Zhang et al., 2006). In MRM, the ability to check assumptions in voxels of interest is readily provided through a number of standard residual plots and inferential tests. Although it is not practical to check every voxel, it should be encouraged to at least check that the model assumptions appear reasonable at peak voxels of interest. Examples of these plots are given later.

### Computational speed

Previous publications discussing multivariate approaches in neuroimaging have commented that the approach can be slow (Chen et al., 2014). As such, there may be concern that the switch from the univariate GLM to the multivariate GLM involves a considerable additional computational burden. Generally speaking, MRM model estimation is fast, making full use of the compiled MATLAB routines for large matrix operations. Using MATLAB R2013a on a 2.3 GHz quad-core i7 MacBook Pro with 16GB of RAM, estimation of 5 dependent variables from 4 groups (a total of 53 subjects and 265 images with dimensions 53 × 63 × 52) takes approximately 17 s. This is inclusive of the estimation and writing of images of the parameter estimates and covariance structure to disc. As such, the only real computational burden is when permutation methods are invoked for inference.

**Fig. 3:**
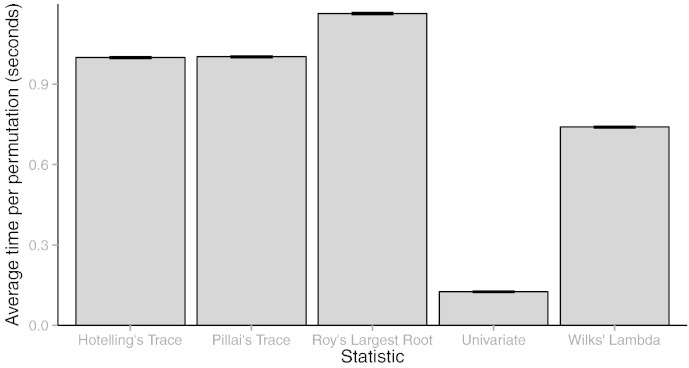
Speed comparisons between the different test statistics taken as an average of 5,000 permutations. The total times, in minutes, to complete all permutations were *Univariate F* = 10.42, *Pillai’s trace* = 83.53, *Wilks’ lambda* = 61.67, *Hotelling-Lawley trace* = 83.27, and *Roy’s largest root* = 96.98. Here the extra computational burden of the multivariate tests is clear. When the tests are exact, Wilks’ lambda is the best choice from a speed standpoint. Error bars represent the standard deviation.

As detailed in Appendix C, the permutation approach in MRM is based on the *randomise* algorithm published in Winkler et al. (2014). For contrasts that simplify to univariate comparisons, this method is fast, generally completing 5000 permutations in around 10 min. For multivariate contrasts, this approach is much slower, compounded by the fact that each voxel no longer represents a scalar but an instance of an SSCP matrix. Although some optimisation of the calculations is possible, the speed of the permutations remains influenced by the number of non-empty voxels in an image and the particular multivariate tests statistic chosen. Fig. 3 demonstrates speed differences for each of 5000 permutations between a univariate contrast and a multivariate contrast using the different test statistics, performed using the hardware detailed above. Generally, Wilks' lambda is the fastest statistic to compute, around 6 × slower than the univariate contrast, with Roy's largest root the slowest at nearly 10 × slower than the univariate contrast. Pillai's trace, as the most robust of the four test statistics, is around 8 × slower than the univariate approach. Unsurprisingly, given their similarity in Eq. (9), Pillai's trace and the Hotelling–Lawley trace are near identical for speed. Further work on integrating GPU computing in neuroimaging software may be able to render processing time for such tests negligible (Eklund et al., 2012).

## Comparisons with existing univariate approaches for repeated measurements

To demonstrate the utility of the repeated-measures aspect of the multivariate GLM, we conducted a number of comparisons between the approach implemented in MRM and univariate approaches to dependent neuroimaging data implemented in other popular MATLAB toolboxes. The software packages chosen for comparison included SPM12 (http://www.fil.ion.ucl.ac.uk/spm/), GLM FLEX (http://mrtools.mgh.harvard.edu/index.php/GLM_Flex), and the recently released Sandwich Estimator (SwE v1.2.2; Guillaume et al., 2014; http://www2.warwick.ac.uk/fac/sci/statistics/staff/academic-research/nichols/software/swe). In our experience, these are the most popular MATLAB packages that researchers use when faced with repeated-measures models of neuroimaging data. We did not conduct comparisons with iterative maximum-likelihood methods as applying such approaches to neuroimaging data has many disadvantages, as discussed by other authors (Chen et al., 2014, Guillaume et al., 2014), including computational burden and uncertainties with respect to the covariance structure that can be sensibly imposed at each voxel. We also did not make comparisons using FSL FEAT given the restrictive necessity of assuming sphericity in order for exact *F*-tests. Excluding FSL FEAT also allowed us to only compare solutions written in MATLAB using SPM functions, allowing for sensible comparisons in terms of speed as well as direct scrutiny and comparison of the MATLAB code, an approach that would be unnecessarily complicated by inclusion of compiled programmes written in languages such as C/C ++.

Both GLM FLEX and SwE use SPM as their base, but expand upon the default mass univariate functions in a number of ways. GLM FLEX allows for the implementation of traditional repeated-measures ANOVA models by allowing the specification of different error terms for each contrast. These error terms are user-specified and are built using the MATLAB scripting interface. In these models, derivation of the correct error term is left up to the user and can be achieved using the expected mean squares of the model (Casella, 2008, Kutner et al., 2005). Importantly for the current comparisons, GLM FLEX uses the SPM non-sphericity modelling procedure^2^ to estimate a covariance structure using restricted maximum likelihood (ReML) on a pooled selection of voxels from an initial model fit. This estimated structure is then used to *pre-whiten* the data in attempt to render the error covariance structure closer to its assumed form (Glaser and Friston, 2007, Poldrack et al., 2011). As mentioned earlier, a key problem with this approach is the assumption that the estimated covariance structure is the same for every voxel in the image. SwE, by comparison, allows for a unique covariance structure to be estimated for every voxel. Similar to the approach in MRM, SwE uses the model residuals at each voxel to estimate a unique covariance structure. This structure is then used to construct ‘robust’ standard errors of the estimated model parameters using a formulation referred to as ‘sandwich’ estimation due to the form that the estimation equation takes (Guillaume et al., 2014). Some of the differences between these packages are given in Table 1.

### Data, preprocessing, and subject-level models

The data used for comparison between the software packages was taken from an investigation into the influence of a history of major depressive disorder (MDD) on affective processing in older and younger adults (McFarquhar, 2015). Twenty-nine younger adults (aged 30–50) and 29 older adults (aged 60–85) were recruited primarily from the Greater Manchester area. All participants completed an initial screening questionnaire followed by a face-to-face clinical interview to assess inclusion and exclusion criteria. The final groups comprised 12 remitted MDD (rMDD) older adults, 12 rMDD younger adults, 14 older adult controls, and 15 younger adult controls. All participants provided informed consent and the study was given a favourable opinion by the local research ethics committee (REC ref. 11/NW/0009).

During the scanning session, participants performed an Affective Go/No-go (AGN) task (Elliott et al., 2000, Elliott et al., 2002, Elliott et al., 2004). In brief, words selected from two categories (e.g. ‘positive’ and ‘negative’) were presented rapidly on a screen in a random order. Participants were instructed to only respond by pressing a button when a word was shown belonging to one category (the ‘target’ category), but not the other (the ‘distractor’ category). Across the task, five variants of the combination of ‘target’ and ‘distractor’ categories were used. This task is therefore a within-subject design due to all participants engaging in all five conditions. There were also two between-subject factors in the investigation consisting of age (older and younger) and diagnostic history (control and rMDD). See Appendix D for details of the scanning parameters.

Prior to group analysis, the data were preprocessed in SPM12 by realigning the images to the first volume, coregistering the structural image to the mean functional image, segmenting the structural image into its constituent tissue classes, applying the estimated transformations to MNI space derived from the segmentation to the functional scans, and finally, smoothing the functional scans using a Gaussian kernel with FWHM of 9 × 9 × 9 mm. As an additional step, we made use of the artefact detection tool (ART; http://www.nitrc.org/projects/artifact_detect/) to identify high motion volumes using a volume-to-volume shift of > 1.5 mm and a volume-to-volume change in mean signal intensity > 3 standard deviations. Any scans with > 20% volumes identified by ART as outliers were excluded. Subject-level models were fit in SPM12 using the HRF + derivatives basis set with the addition of the per-subject regressors produced by ART to ‘censor’ high-motion volumes (Power et al., 2012, Siegel et al., 2014). Specifically, we modelled the five conditions of the task leaving the rest periods as an implicit baseline. As such, there were five parameters, one per condition, that were taken to the group level from each participant.

### Group-level modelling approach

To allow for maximum comparability between the software packages, a number of restrictions were placed on the initial modelling procedure (see below for comparisons with these restrictions lifted). Firstly, we assumed that the covariance structure of the data was homogenous across the groups. This involved setting the group variance options to ‘equal’ in SPM and GLM FLEX, and defining only a single covariance matrix group in SwE. This is presently the only option in MRM. Secondly, we restricted thresholding to an uncorrected *p* < 0.01 in an effort to best visualise the differences between the packages, foregoing any *p*-value correction in an effort to rule out any differences due to implementation of correction techniques across the software.

**Fig. 4:**
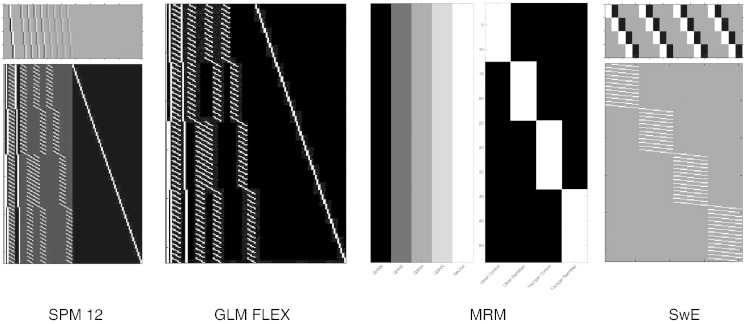
The different design visualisations from the four software packages. It is notable than until very recently (update 6470 for SPM12) the design shown above would not have been possible given limitations previously imposed on the SPM Flexible Factorial module. The contrast for the main effect of the repeated-measurement conditions is displayed visually above the design matrices in both SPM and SwE. The fractional contrast weights visible above the SPM design matrix are a consequence of constructing estimable functions in overparameterised designs. In MRM the factorial structure of the outcome matrix is shown visually as shaded columns to the left of the design matrix.

Using these restrictions, we estimated a classical repeated-measures ANOVA model using the SPM Flexible Factorial module for the within-subject main effects and interaction tests, and a second between-subject ANOVA model averaging over the repeated-measurements for the between-subject main effects and interaction tests. Although previous authors have suggested that SPM incorrectly estimates between-subject effects in repeated-measures models (Chen et al., 2014, McLaren et al., 2011), this is only true when no concern is given to the error term for the tests. This is an issue that has a long history in the analysis of split-plot designs (Casella, 2008, Christensen, 2011), where the issue of *error strata* has been thoroughly discussed for those situations when a random effect (e.g. *subject* in a repeated-measures design) is included in the GLM (Nelder, 1977). Here we include the comparisons with SPM in part to show that it *is* possible to fit these models correctly in SPM. How easy this is to do, however, is a different question given that multiple models are often needed, and that in the Flexible Factorial module contrasts need specifying as estimable functions in an overparameterised linear model framework^3^ (Green et al., 1999). In GLM FLEX, SwE, and MRM, only a single model was needed to correctly estimate all comparisons of interest. In GLM FLEX, features such as accommodating missing voxel-level data across subjects and outlier detection were switched off in order to facilitate comparisons. In terms of the extra options available in SwE, we specified the small sample adjustment as type ‘C2’, as this is the recommended option in the software for the most accurate bias correction. The calculation of the degrees of freedom was set to use the ‘approximate II’ approach as, similar to the small sample adjustment, this is recommended by the authors of the toolbox as the most accurate approach when there is no missing data. In MRM, Wilks' lambda was used as the test statistic. Because the comparisons in this example were exact, this choice made no difference to the results. Fig. 4 shows the model setup from all four software packages. Here we display the full factorial design in SPM for maximal comparability with the other design. It should be noted, however, that not all the presented columns are strictly necessary to form the tests of interest and that tests of any pure between-subject effects in this model would not be suitable as the error term derived from the model residuals would be incorrect.

### Results

**Fig. 5:**
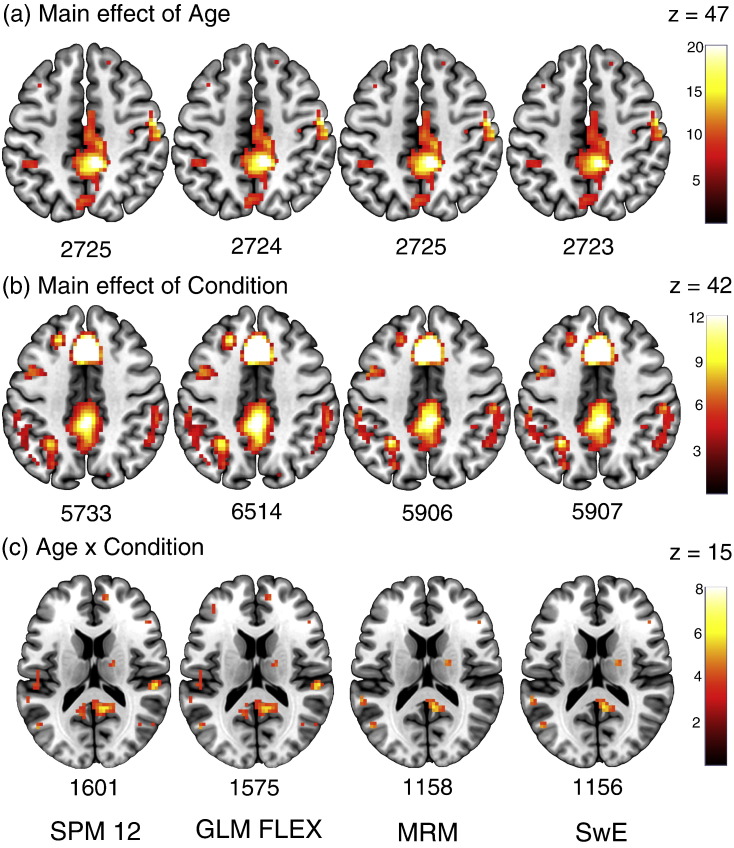
Examples of the results found for 3 different contrasts from the 4 software packages. (a) The main effect of age (b) The main effect of the repeated measurement conditions (c) The age by repeated measurement condition interaction. All contrasts are thresholded at *p* F-values. Note these results come from the restricted model comparisons where covariance homogeneity is assumed.

Fig. 5a shows the results across the four software packages for the main effect of age contrast. Of note is the fact that SPM and MRM are identical. This is as expected given that the multivariate GLM simplifies to univariate form under between-subject comparisons. Although identical in principle, the results from GLM FLEX differ from both SPM and MRM. This appears to be a result of differing implementations of the SPM non-sphericity correction and the subsequent whitening that is applied to the design. As demonstrated in Appendix E, this has direct consequences for the parameter estimates from the model, leading to the discrepancy in the calculated test statistics. Because the SPM between-subject comparisons are performed using a model where the repeated measures have been averaged, and because we assume covariance homogeneity in this example, no whitening will have been applied. This leads to identical results in SPM and MRM. This is not true in GLM FLEX, as the between-subject comparisons are performed within the same model as the within-subject comparisons. This means any whitening applied due to the repeated measures has the potential to also impact the between-subject comparisons. SwE, on the other hand, appears the more conservative of the approaches. That being said, results are so similar across the software packages that this would be of little practical significance.

**Fig. 6:**
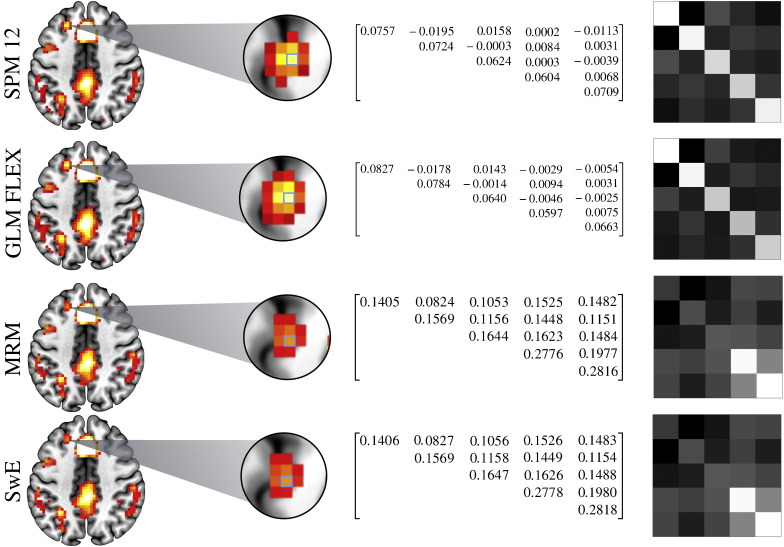
Comparison of the within-subject covariance estimates from the four software packages in the highlighted voxel (−21 29 44 in the main effect of condition). Because of subtle differences in the estimated values between all the software packages each matrix is presented as a scaled image on the far right. Both MRM and SwE save the covariance estimation to images. SPM and GLM FLEX keep the global covariance calculation used in the pre-whitening in their respective *.mat files (e.g. SPM.xVi.V). These matrices were extracted and then scaled by the individual voxel variance. These values can then be used to calculate $\hat{\beta }$ and $\left(\hat{\text{Var}}\right)$$\left(\hat{\beta }\right)$ using a generalised least squares scheme, providing identical results to the pre-whitening approach (see Faraway, 2005, p. 89; Poldrack et al., 2011, p. 196). Note these results come from the restricted model comparisons where covariance homogeneity is assumed.

The contrast for the main effect of the repeated-measurement conditions is shown in Fig. 5b. Here, a number of differences between the methods become apparent. Firstly, although largely similar, there are again differences between the thresholded maps provided by SPM and GLM FLEX. In this example, GLM FLEX appears to be generally estimating larger *F*-values than SPM, leading to the discrepancy in the number of voxels that survive thresholding. As an example, the result from 0 20 47 is given as *F*_4,196_ = 41.35 in SPM and *F*_4,196_ = 45.84 in GLM FLEX. Again, this appears to be a consequence of differing implementations of the SPM non-sphericity correction, and is explored in more detail in Appendix E. The results from MRM and SwE, on the other hand, appear largely comparable, with SwE slightly more sensitive. Of particular interest is comparing SPM/GLM FLEX to MRM/SwE given that the former methods choose to pool the covariance estimate whereas the latter estimate a unique covariance matrix per-voxel. There are a number of regions in these maps where, despite subtle differences in their estimates, SPM and GLM FLEX have generally provided a larger test statistic value compared with MRM and SwE. Because these differences are consistent with the different methods of covariance estimation, this may suggest that the pooled approach is artificially inflating the test statistic at certain voxels. This point is further explored in Fig. 6 where an example voxel is compared in terms of the estimated covariance structure across the different software packages.

As a final comparison, the age × condition interaction is shown in Fig. 5c. A similar result to the main effect of condition is evident here. Again, subtle differences in the calculated statistics are present in SPM and GLM FLEX; however, the discrepancy between the pooled covariance approaches and the unique covariance approaches is again clear in the SPM/GLM FLEX and MRM/SwE divide. Both SPM and GLM FLEX appear more sensitive, although whether this is due to differences in power (because of differences in the number of parameters each method must estimate) or differences in the estimated covariance structure is unclear. Again, MRM and SwE largely agree, with SwE the slightly more conservative of the two.

### Unrestricted model comparisons

**Fig. 7:**
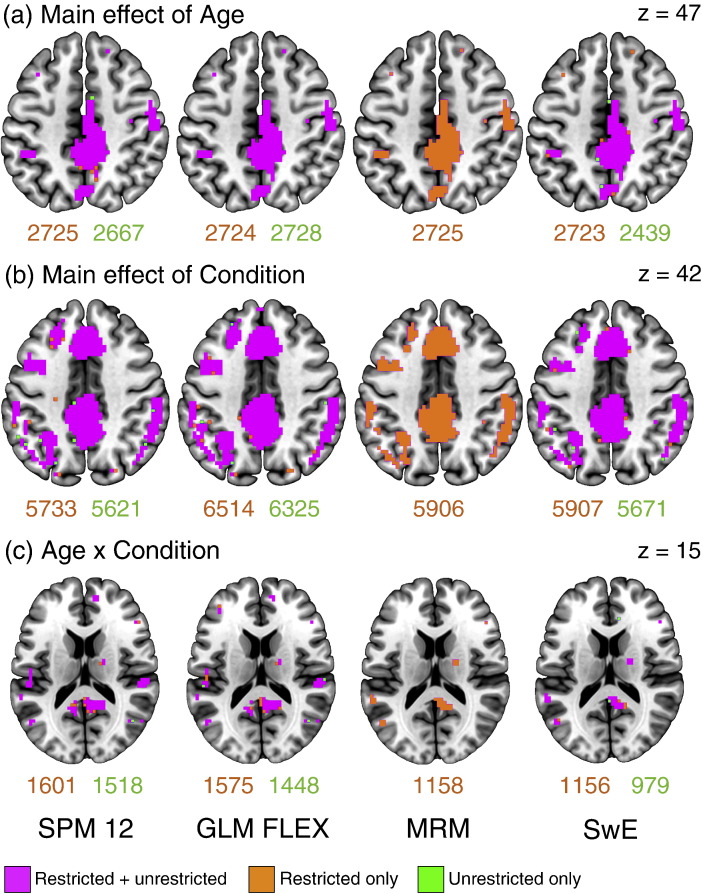
Comparisons between the restricted and unrestricted models in each of the software packages. Voxels in pink indicate those that survive thresholding in both the restricted and unrestricted models. Voxels in orange are those that survive thresholding in the restricted model only, with voxels in green showing the same for the unrestricted model. The values beneath the images indicate the number of voxels that survive thresholding in the restricted and unrestricted models. For SPM, GLM FLEX, and SwE the unrestricted models equate to estimating a unique covariance structure per-group. As MRM assumes covariance homogeneity, only the restricted results are presented.

To further compare these approaches, we estimated the models in each of the software packages using fewer restrictions. For SPM 12 and GLM Flex, this involved setting the group variances to unequal. For SwE, this involved requesting a unique covariance matrix to be estimated for each group. As previous authors have demonstrated, assuming covariance homogeneity when the reality is heterogeneity can lead to either conservative or liberal inference (Guillaume et al., 2014). It is therefore important for researchers to realise the potential limitations of making this assumption in the multivariate GLM. Fig. 7 shows the comparisons between the models estimated earlier and those estimated with fewer assumptions. Voxels in *pink* indicate overlaps between the previous model and the unrestricted model. Voxels in *orange* indicate those found in the restricted model only, with voxels in *green* indicating those found in the unrestricted model only. Looking across these results, it is clear that although the number of voxels surviving thresholding do differ between the restricted and unrestricted models, these are generally fringe cases on the edges of clusters that appear irrespective of the covariance assumptions. In addition, it is also clear that assuming covariance heterogeneity generally leads to more conservative inference, and while this is preferable to overly liberal inference, it will lead to a reduction in power if homogeneity can be assumed. This appears particularly true of SwE, where the reduction in surviving voxels when covariance heterogeneity is assumed is consistently the greatest. Again, SPM and GLM FLEX appear to differ due to their implementations of the non-sphericity correction, with the SPM/GLM FLEX and MRM/SwE split still apparent. This would suggest that the biggest differentiator between these methods is not their ability to accommodate a different covariance structure per group; rather, it is their use of unique vs pooled structures across an image. As such both MRM and SwE are the preferred approaches, with SwE providing more flexibility in allowing the covariance structure to differ between groups, but seemingly losing some sensitivity, particularly in the between-subject comparisons. It is also worth noting that the multivariate approach is capable of incorporating covariance heterogeneity using approximate degrees of freedom corrections such as the Welch–James and Brown–Forsythe approaches (Keselman and Lix, 1997, Lix et al., 2003, Vallejo et al., 2001). These are, generally speaking, more complex to implement than the standard multivariate test statistics, and given that they are not widely used, they will require further investigation before applying to imaging data. Presently, assumptions of covariance homogeneity can be checked in MRM at peaks of interest, allowing researchers to caution interpretation if this assumption appears violated.

### Assumption checking

**Fig. 8:**
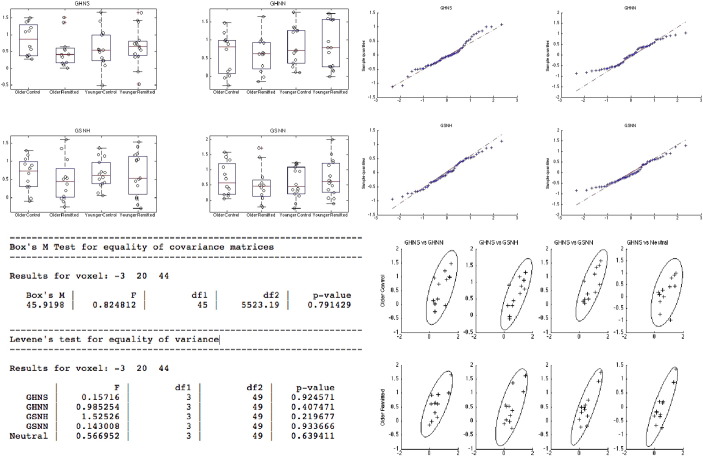
Examples of the assumption checking procedures in MRM. Here we display box and jittered scatter plots of the raw data, QQ plots of the residuals, the results from Box’s and Levene’s tests, and scatter plots of the raw data in dependent variable pairs for each group (contour lines represent 2 standard deviations of the implied bivariate normal distribution). For Levene’s test, the acronyms for the different conditions of the AGN are as follows: GHNS = “go happy no sad”, GHNN = “go happy no neutral”, GSNH = “go sad no happy”, and GSNN = “go sad no neutral”. Note to save space only a subset of the full plots is shown.

One advantage of the MRM software is the ability to check model assumptions at peak voxels of interest. These checks include both standard inferential tests of the model assumptions, as well as a wealth of plotting devices that allow the researcher to assess the plausibility of the model at voxels of their choosing. Though assumptions of multivariate normality can be relaxed when using non-parametric permutation methods, the plausibility of this assumption can still be checked using quantile–quantile plots of the model residuals. Here, we follow the recommendations of Christensen (2001) and provide plots of the residuals for each dependent variable separately, as well as a single linear combination (the sum). For covariance homogeneity, we provide both Box's *M* test (Box, 1949, Box, 1950), and, again following from Christensen (2001), plots of dependent variable pairs for each cell of the design. For the moment, researchers are encouraged to exercise caution and use Pillai's trace as the test statistic in cases where this assumption appears violated. For between-subject comparisons, we similarly provide Levene's test for homogeneity of variance (Levene, 1960), as well as plots of the fitted values against residuals. Examples of some of these plots and tests are given in Fig. 8 for a peak voxel from the main effect of condition contrast. Results from these checks imply that the assumption of covariance homogeneity appears reasonable at this voxel. There is some suggestion of violations of normality in the tails of the distribution, and as such, we may wish to use permutation approaches to calculate *p*-values for the hypothesis tests. It is also worth noting that such tests can easily be applied to univariate group-level neuroimaging data in MRM by setting the number of within-subject factors to 0 (or the number of dependent variables to 1), allowing MRM to be used as a more generic group-level modelling tool.

### Comparison of approaches to FWE correction

**Fig. 9:**
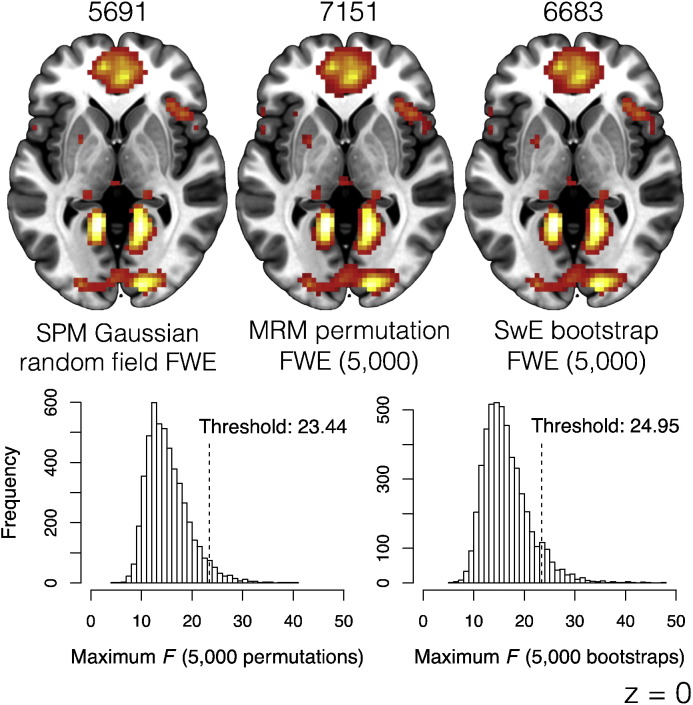
Comparisons between the SPM Gaussian random field, MRM permutation, and SwE non-parametric bootstrap approaches to FWE correction. The numbers above the images indicate the number of voxels surviving thresholding in each image. The permutation and bootstrap distributions are displayed with the 5% thresholds indicated. The original maximum reference test statistic has been cropped to make the histograms more readable. For comparison, the 5% *F* threshold given by SPM12 was 28.40. Total time to complete the non-parametric approaches (in minutes) were MRM = 12.62, SwE = 213.88.

Though the utility of the multivariate framework for dependent data has been demonstrated, it may be of some concern that the methods used for FWE correction in this framework do not make use of the standard neuroimaging approach provided by GRF. As a final comparison, we investigated the difference between the GRF FWE correction and the FWE correction resulting from permutation tests. We also included comparison with the non-parametric bootstrap option provided in SwE. We compared the main effect of task from the SPM model using GRF FWE correction, from the MRM model using permutation-based FWE after 5000 permutations, and the SwE model using bootstrap-based FWE after 5000 bootstraps. As both permutation and bootstrap approaches depend on the number of model re-fits to dictate the precision of *p*-value estimation this allowed both non-parametric approaches to calculate values in the interval 0.0002 ≤ *p* ≤ 1. Fig. 9 shows the comparison between the results as well as the permutation and bootstrap distributions of the maximum *F* in the image. Table 2 shows comparisons between the *p*-values for the seven smallest peaks reported by SPM. Generally, results are similar across the approaches, with the GRF method providing larger *p*-values in all cases. Both the permutation and bootstrapping approaches lead to very similar estimated null-distributions and subsequent 5% thresholds. In this example, the MRM permutation approach is slightly more liberal than the bootstrapping approach. These results may therefore suggest that the GRF approach to voxel-level statistics is overly conservative, consistent with results presented previously by Nichols and Hayasaka (2003) and more recently by Eklund et al. (2015).

## Comparisons between the multivariate test statistics

**Fig. 10:**
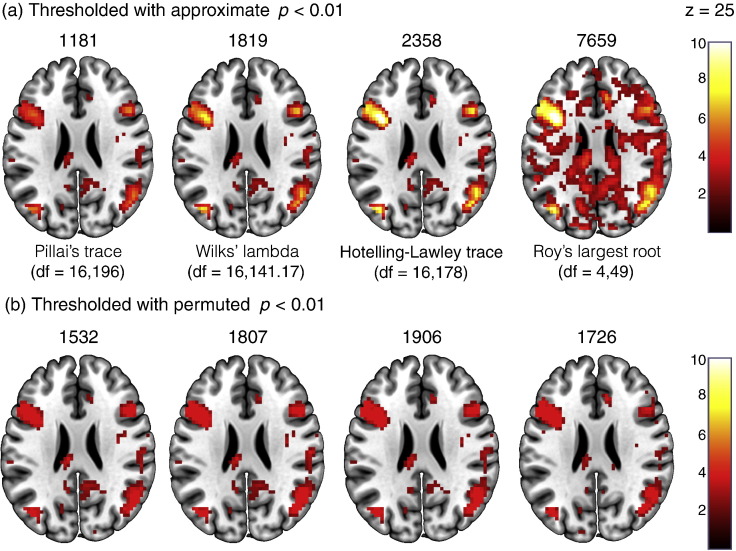
Comparisons between the four different multivariate test statistics for a non-exact multivariate test (a) thresholded at *p* p-value approximations and (b) thresholded at *p*p-values derived from 5,000 permutation tests. Results are presented as *p*-values transformed using –log_10_. The numbers above each image indicate the number of voxels that survive thresholding.

As indicated earlier, when using the multivariate GLM, there is a choice of four potential test statistics. Such a choice provides greater complexity to the use of the multivariate GLM in neuroimaging when using contrasts that produce non-exact *F* values. Though these tests have been compared numerous times in the statistical literature (Ito, 1962, Lee, 1971, Mikhail, 1965, Olson, 1974, Pillai and Jayachandran, 1967), we sought to briefly investigate their behaviour when applied to real neuroimaging data. To do this, we used the **C** matrix from the main effect of condition contrast detailed earlier with **A** = **I**_*k*_. We compared both the approximate *p*-values associated with the different test statistics as well as the *p*-values derived from 5000 permutations. Fig. 10a shows the results for the classical *p*-value approximations, with the test statistics displayed from most conservative to least conservative. Here, the nature of Roy's largest root as an upper-bound on the *F*-value is clear. Pillai's trace, Wilks' lambda, and the Hotelling–Lawley trace are all similar, with the Hotelling–Lawley trace the most liberal, and Pillai's trace the most conservative. These results agree with previous recommendations suggesting Pillai's trace is the safest test to use as it provides the best control over type I errors. These results also suggest that the *F* approximation to Roy's largest root should generally be avoided unless there is good reason to only consider the upper-bound. In Fig. 10b, we present the same comparisons thresholded using *p*-values derived from permutation testing. Because we only ran 5000 re-shuffles the largest possible value in the map is -log_10_1/5000 = 3.70. What is noticeable is that for Roy's largest root, the pattern of results is much more in keeping with the activation maps found for the other test statistics. The permutation approach therefore appears to converge the behaviour of the test statistics as under permutation the *p*-values of Roy's largest root no longer represent an upper-bound, rather they more closely reflect the true *F*. In addition, it is interesting to note that in this example, Wilks' lambda appears the most consistent between the approximate and permutation-based *p*-values. This suggests that, although not necessarily generalisable to every dataset and contrast, when using permutation approaches, the differences between the test statistics may be less of a concern and the choice can be driven more by the computational considerations discussed earlier.

## Multimodal integration

Although much detail has now been given about the repeated-measures aspect of the multivariate GLM, we have yet to demonstrate its utility in the integration of multimodal and multi-sequence imaging data. To do so, we present a combined voxel-based morphometry (VBM) and functional MR analysis using the younger adults of the sample described earlier. Specifically, we sought to compare those with a history of depression to those with no history of depression under the condition of viewing negative images. The task performed by the participants was based on a memory paradigm reported in Whalley et al. (2009), but was altered to include negative images alongside the originally reported positive and neutral. Seventy-two images were selected from the International Affective Picture System (IAPS; Lang et al., 2008) comprising 24 positive, 24 negative, and 24 neutral. Stimuli were presented in blocks of 6 images from one valence category. A period of rest was provided for 15 s after each cycle of positive, negative, and neutral blocks. During the task, 145 volumes were collected, with all other scanning parameters identical to those described in Appendix D.

### Image preprocessing

For the functional scans, preprocessing was conducted in an identical fashion to the procedure described earlier. Again, subject-level models were estimated in SPM 12 using the HRF + derivatives basis set with the addition of the per-subject regressors produced by ART. Here, we modelled the three picture conditions of the task again using the rest conditions as an implicit baseline. Unlike the demonstration earlier, only the parameter estimates associated with the *negative* image condition were taken to the group modelling stage. For the structural scans, the SPM DARTEL tools (Ashburner, 2007) were used to produce normalised grey matter images using the segmented tissue images from the preprocessing.

One particular issue in using the MANOVA approach for modelling multimodal data is that different modalities often provide images of different resolution. For example, a typical structural MR image may have around 10 times as many voxels as a typical task-based fMR image. In order for a voxel-by-voxel analysis to work, it is necessary to rescale one of the modalities to match the other. Our own limited investigation of this issue suggests that results are relatively invariant to whether one up-samples the functional to the dimensions of the structural, or down-samples the structural to the dimensions of the functional. Where the benefit of resampling the higher resolution image becomes clear is with the increase in computational speed and decrease in computational burden for model estimation and inference by permutation, as well as a reduction in the number of hypothesis tests that must be corrected for at the voxel-level. That being said, the choice of approach will likely depend on the modality of most interest, and the investigator's opinion on the trade-off between increased computational speed and the loss of information engendered by interpolating a higher-resolution image to smaller dimensions.

Another issue, typical to VBM investigations, is the necessity of a correction for head size to allow for sensible between-subject comparisons. In SPM, it is possible to provide values to perform *proportional scaling* of the images before the model is estimated. As there is no such facility in MRM, the proportional scaling was performed manually on the normalised grey matter images before they were entered into the model. Specifically, the value at each voxel of the normalised grey matter images was divided by the participant's total intracranial volume (estimated using the Easy_volume tool http://www.sbirc.ed.ac.uk/cyril; as described in Pernet et al., 2009) to produce proportionally scaled versions of the DARTEL results. For the multivariate GLM, this strategy is preferable to entering these values as covariates given that any covariate will influence *all* the model parameters, irrespective of the modality. This could be seen as a disadvantage of the multivariate approach to multimodal integration, particularly in those cases where co-varying for a nuisance variable in one modality is seen as preferable to rescaling the raw data. Other covariates that may be relevant for both modalities can be entered into the model directly, though for simplicity of presentation, we do not include any here. Only after the proportional scaling were the grey matter images resampled to the same dimensions as the images of parameter estimates from the functional models. In addition, it is worth mentioning that at present the permutation approach implemented in MRM does not account for non-stationarity when using cluster-level inference. As approaches to permutation that accommodate non-uniform smoothness of the images have been proposed by Hayasaka et al. (2004), this could be implemented in the future to allow researchers to appropriately use cluster-level statistics for analyses of data such as structural MR images.

As a final step, we produced a mask to restrict the analysis to only regions of grey matter. This was done by averaging the scaled and resampled grey matter images and then producing a binary image including voxels with an intensity > 0.2. Such an approach is in keeping with the recommendations given by Ashburner (2010).

### Model estimation and results

The group-level model used for these data consisted only of the grouping variable for controls or rMDD. The model was therefore equivalent to a multivariate form of a simple two-sample *t*-test. As this design was specified as a MANOVA model, the structural and functional data were treated as non-commensurate. As such, the **C** matrix of the general linear hypothesis test of the main effect of diagnosis was specified as **I**_2_. Results of this contrast, thresholded at an uncorrected *p* < 0.001, revealed a cluster of 48 voxels in the left lingual gyrus (peak at − 15 − 73 − 7 with *F*_(2,25)_ = 19.26). Following up this result using *d*LDA at the peak voxel revealed a single discriminant function with absolute values of the standardised weights given as 0.826 for the structural modality and 0.850 for the functional modality. This result is particularly interesting because it suggests that, at this peak voxel, a near equal balance of the modalities provides maximal separation of the groups. Using the partial *F*-test methodology described in Appendix B gives significant results for both the structural and functional modalities (both *p* < 0.001), suggesting that each modality is contributing to group separation.

**Fig. 11:**
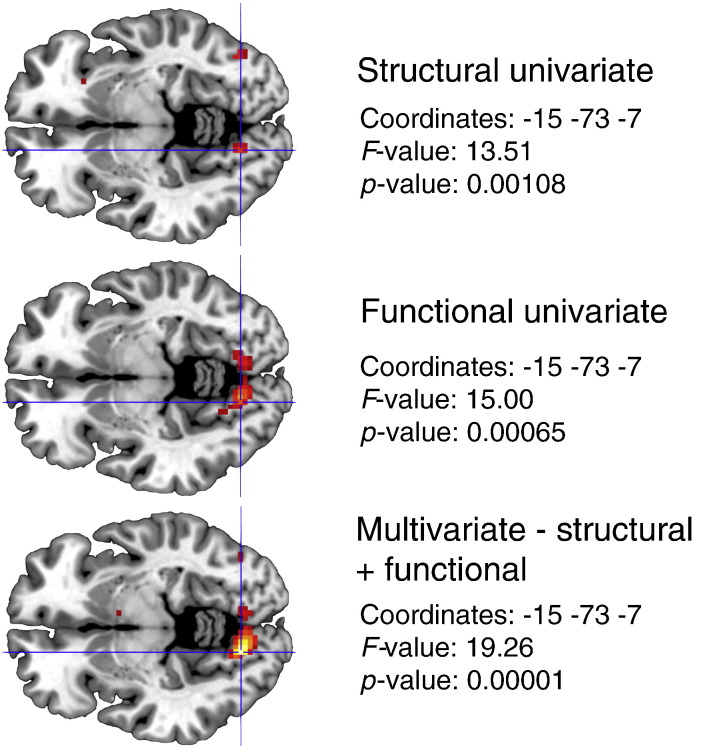
Comparison between the univariate VBM analysis (top), the univariate fMRI analysis (middle), and the multivariate GLM approach integrating both modalities (bottom). Images have been thresholded at an uncorrected *p* < 0.01 for display purposes.

Of further interest here is that conducting the univariate equivalents of this analysis on each modality separately revealed smaller test statistics at this peak, as shown in Fig. 11. Here, a clear advantage of the multivariate approach is seen as the individual results from the univariate analyses have been strengthened by virtue of the fact that equivalent results are seen across modalities. The results of the *d*LDA at this voxel enhance this interpretation given that nearly equal weight is given for each modality. Although thresholded liberally for our demonstration, these findings suggest that the multivariate approach has the scope for providing true integration of functional and structural information in a single model, allowing researchers to investigate those regions where the information across modalities can be effectively pooled to maximally discriminate between groups of interest.

## Limitations of the multivariate GLM for neuroimaging

Although there are clearly many advantages to the multivariate GLM for group-level analyses of neuroimaging data, there remain a number of drawbacks. Perhaps most problematic are times when the power of the multivariate approach is limited compared with univariate methods due to the number of parameters it must use (Davis, 2002). Indeed, it is possible that for some models a more parsimonious number of parameters could be estimated when using an alternative univariate framework, allowing certain questions to be more easily addressed using an alternative modelling scheme. This is particularly true of the *p*-block approach to pharmacological challenge MRI (phMRI; e.g. McKie et al., 2011) where the number of time bins is more severely limited in the multivariate framework compared to the univariate. Similarly, there are also limitations in the number of subjects necessary for an analysis compared with the number of repeated-measurements or modalities (Chen et al., 2014). The current assumptions of covariance homogeneity may also prove problematic for instances of multiple groups, particularly when the data are severely unbalanced and the robustness of the multivariate test statistics can no longer be guaranteed. Here permutation approaches may help, but are certainly not guaranteed solutions when faced with arbitrary violations of the parametric assumptions (Finch and Davenport, 2009). The integration of time-varying covariates into the model is also not possible. In addition, the fact that for multimodal models any continuous covariate may influence estimates for both modalities can also be seen as a disadvantage, reducing the flexibility of this approach to deal with confounding factors specific to one modality but not the other. Finally, it is not possible to incorporate subjects who have missing data on any of the modalities or repeated measurements. Although generally not problematic when modelling conditions of a task, missing data may be an issue for longitudinal designs with significant subject attrition. This is one advantage of the SwE approach over MRM as within-subject missing data can be more readily accommodated using the sandwich estimator framework. For well-powered investigations with no missing repeats and no critical time-varying covariates, we believe that the multivariate approach is one of the most straightforward method of analysing the data given that it a simple extension of the existing univariate GLM. Indeed, given that the approach is not restricted to only repeated measurements, and easily simplifies to univariate GLM analyses identical to those already in use in neuroimaging, we argue that the multivariate GLM is the most generic and conceptually straightforward approach to dealing with dependent neuroimaging data.

## Summary

In this paper, we have provided an exposition of the use of the multivariate GLM in neuroimaging applications specifically as a method for analysing both repeated measurement and multimodal imaging data at the group level. We have explored methods of making inference in these forms of models and have shown comparable results to other approaches to dependent imaging data. Furthermore, the use of this approach combined with *d*LDA for multimodal investigations opens up a wealth of possibilities for integrating different imaging tools to better understand distinctions between groups of interest. We believe the multivariate approach is ideal for application to neuroimaging data due to its computational speed, straightforward hypothesis-testing framework, and minimal assumptions. The MRM software is free to download from The University of Manchester's Click2Go service at http://www.click2go.umip.com/i/software/mrm.html.

## Figures and Tables

**Table 1:** Comparison between a number of the features pertinent to repeated-measures models available in the four software packages.

Software	GUI	Voxel-level		Cluster-level		Unique voxel covariance	Unequal group covariance
FWE correction	FDR correction	Cluster size	Cluster mass
SPM 12		_1_	_4_				
GLM FLEX		_1_					
MRM		_2_					
SwE		_3_					

^1^Using Gaussian random field theory.

^2^Using permutation testing.

^3^Using a non-parametric bootstrap.

^4^Setting topoFDR = 0 in spm_defaults.m.

**Table 2:** *P*-value comparisons between the different FWE methods for the seven smallest maxima reported by SPM.

Peak location (mm)			*p*-GRF	*p*-PERM	*p*-BOOT
x	y	z
− 45	− 37	17	0.002	< 0.001	0.001
42	− 16	35	0.008	0.003	0.004
0	− 25	− 1	0.019	0.006	0.007
21	59	5	0.026	0.008	0.011
− 33	− 1	− 37	0.035	0.010	0.015
− 21	35	38	0.035	0.010	0.015
51	11	2	0.047	0.011	0.020

*p*-GRF = FWE-corrected *p*-values from the SPM GRF approach.

*p*-PERM = FWE-corrected *p*-values from the MRM permutation approach (5000 reshuffles).

*p*-BOOT = FWE-corrected *p*-values from the SwE non-parametric bootstrap approach (5000 bootstrap resamples).

## Appendix A The choice of multivariate test statistics

As detailed in the text, there are four potential tests statistics that can be used with the multivariate GLM. The natural question of which of the four test statistics should be chosen is somewhat problematic as the answer largely depends upon the data itself. Because of this, it is not uncommon for statistical software to provide all four. For example, when the population eigenvalues are roughly equal, Pillai's trace is the most powerful test, whereas when they are unequal, the Hotelling–Lawley trace is the most powerful test (Davis, 2002). These values are never known in practise, as they are population parameters, making decisions between the tests difficult. Luckily in many circumstances, the *F* approximation to these tests are exact and will therefore be identical irrespective of the test chosen. Although the conditions under which exact tests are produced differ between the four statistics (see Rencher and Christensen, 2012 for details of each), all the tests will be exact when rank(SSCP_*h*_) = 1. This can be tested for within MRM. When the tests are not exact, decisions can be made based on the power and the robustness of the test. For example, Davis (2002) recommends Wilks' lambda on the basis that its power remains relatively consistent across simulation studies. Pillai's trace, however, is often quoted as the most robust of the four tests (Olson, 1974) and as such is often recommended when parametric assumptions are not sufficiently met (Tabachnick and Fidell, 2007). Of all the test statistics, Roy's largest root is the most liberal (as it is based solely on *λ*^⁎^) and as such carries with it the greatest risk of a type I error (it is an upper bound on the *F*-value; Rencher and Christensen, 2012). Despite this, some authors support the utility of this test statistic, arguing for its characterisation of the ‘optimal’ linear combination of the dependent variables (Harris, 2013, pp. 231–233). Indeed, for those cases where the largest eigenvalue dwarfs all others, it can be argued that our interest should only be on this single dimension, and Roy's largest root would be the most appropriate. For further discussion on the differences between these test statistics, see Anderson (1984); Davis (2002), and Rencher and Christensen (2012).

## Appendix B *d*LDA details

As described in the main text, the *d*LDA model is given by

(B.1) $$z_{\mathrm{ij}}=a_{1}y_{\mathrm{ij}1}+a_{2}y_{\mathrm{ij}2}+\cdots +a_{t}y_{\mathrm{ijt}}=a^{'}Y_{\mathrm{ij}}$$

Values for **a** can be found using the eigenvectors, and associated eigenvalues, of ${{\mathrm{SSCP}}_{E}}^{-1}{\mathrm{SSCP}}_{H}$ (Klecka, 1980, Rencher and Christensen, 2012). Because ${{\mathrm{SSCP}}_{E}}^{-1}{\mathrm{SSCP}}_{H}$ is generally not symmetric, the calculation of eigenvalues and eigenvectors is performed using the Cholesky factorisation of **SSCP**_*E*_ (see Rencher and Christensen, 2012, p. 290). Though not necessarily an issue for the standard multivariate statistics (as all but Roy's largest root have a form that does not depend on direct calculation of the eigenvalues), for *d*LDA, it is therefore a requirement that **SSCP**_*E*_ is positive-definite. In this scheme, **SSCP**_*H*_ reflects the MANOVA null hypothesis of no group differences. The use of ${S{\mathrm{SCP}}_{E}}^{-1}{\mathrm{SSCP}}_{H}$ is a generalisation of $\left({\overset{-}{z}}_{1}-{\overset{-}{z}}_{2}\;\right)/s_{z}$ to accommodate more than two groups. This generalisation provides a useful indication of the connection between the MANOVA and *d*LDA approach as in the MANOVA approach it is the *q* eigenvalues (*λ*_1_, … , *λ*_*q*_) of ${{\mathrm{SSCP}}_{E}}^{-1}S{\mathrm{SCP}}_{H}$ that form the test statistics, whereas in *d*LDA, it is the associated *q* eigenvectors (**v**_1_, … , **v**_*q*_) that provide information on group separation. The number of non-zero eigenvalues, and therefore the number of discriminant functions, is *q* = rank(**SSCP**_*H*_). Although there may be multiple functions, it is generally the case that one or two are enough to explain the majority of variance. This can be formalised by examining the percentage $\left(\lambda_{s}/\sum_{i=1}^{q}\lambda_{i}\right)\times 100$ for eigenvalue *s*.

In terms of the discriminant functions themselves, it is typical to scale the eigenvectors by $\sqrt{n-k}$ to form unstandardised discriminant functions. Although possible to use the raw **v**_*s*_ vectors the values of *z*_*ij*_ given by these weights are not immediately interpretable. Scaling each vector by $\sqrt{n-k}$ allows *z*_*ij*_ to be interpreted as the distance, in standard deviation units, of each case from the *grand centroid* of the groups (Klecka, 1980). The weights of discriminant function *s* are therefore given by $a=v_{s}\sqrt{n-k}$ with constant $a_{0}=-\sum_{p=1}^{t}a_{p}{\overset{-}{y}}_{p}$, where *a*_*p*_ is the *p*th element of **a** and ${\overset{-}{y}}_{p}$ is the mean of dependent variable *p*. Although these weights provide more interpretable *z*_*ij*_ values, they still relate to dependent variables on their original scale. For dependent variables that are not commensurate, it is generally more useful to interpret the values of the discriminant function when the weights relate to standardised variables. To achieve this, *standardised* discriminant functions are calculated. Defining $M=\text{diag}\left(\sqrt{{{\mathrm{SSCP}}_{E}}_{11}},\sqrt{{\mathrm{SS}{\mathrm{CP}}_{E}}_{22}},\ldots ,\sqrt{{\mathrm{SSC}P_{E}}_{\mathrm{tt}}}\right)$, we use **a**^⁎^ = **v**_*s*_^'^**M** to scale the individual elements of **v**_*s*_. The standardised weights given in **a**^⁎^ are therefore those associated with the following function:

(B.2) $$z_{\mathrm{ij}}=a_{1}^{*}\frac{y_{\mathrm{ij}1}-{\overset{-}{y}}_{i1}}{s_{1}}+\cdots +a_{p}^{*}\frac{y_{\mathrm{ijp}}-{\overset{-}{y}}_{\mathrm{ip}}}{s_{p}}+\cdots +a_{t}^{*}\frac{y_{\mathrm{ijt}}-{\overset{-}{y}}_{\mathrm{it}}}{s_{t}}$$

where ${\overset{-}{y}}_{\mathrm{ip}}$ is the mean of group *i* associated with dependent variable *p*, and *s*_*p*_ is the standard deviation for dependent variable *p*. It should be clear that these are simply the weights associated with dependent variables that have been *z*-transformed.

Although the aim of *d*LDA is largely descriptive, it may be desirable to perform hypothesis tests on the functions. Note that in order to do so, multivariate normality must be assumed. This was not necessary for the calculation of the discriminant functions themselves, only the assumption that the covariance matrices were constant over groups. To perform tests on the individual discriminant functions, Wilks' lambda can be used on a subset of the eigenvalues. For *m* discriminant functions, a sequential elimination procedure can be used, where for each *m*th step the test statistic is

(B.3) $$\Lambda_{m}=\prod_{i=m}^{q}\frac{1}{1+\lambda_{i}}$$

The *F* approximation given in Rencher & Christensen (2012, p. 296) allows for designation of an approximate *p*-value which, if significant, allows one to conclude that *at least* function *m* is significant. With regards to interpreting the contribution of each dependent variable to group separation, one can move beyond examining the absolute values of the coefficients (though this is usually the most informative method) to calculating partial *F*-tests for each of the *t* dependent variables. Here, again, we make use of Wilks' lambda for testing a single dependent variable after adjusting for all other dependent variables. This is done by calculating a *partial* Wilks' lambda, which for the *p*th column of **Y** is given by

(B.4) $$\Lambda \left(Y_{p}|Y_{1},\ldots ,Y_{p-1},Y_{p+1},\ldots ,Y_{t}\right)=\frac{\Lambda_{t}}{\Lambda_{t-1}}$$

where *Λ_t_* is the test statistic from the model containing all *t* dependent variables, and *Λ_t_*_-1_ is the test statistic from the model where the *p*th dependent variable has been removed. This can be interpreted as the contribution of the *p*th variable to group separation above and beyond the contribution of the remaining *t*-1 variables. Again, using the *F* approximation given by Rencher & Christensen (2012, p. 300) allows designation of an approximate *p*-value. Of note is the fact that the partial *F*-tests given here do not relate to a single discriminant function, rather they are a general index of the contribution of the *p*th dependent variable to group separation across dimensions. For models with a single function, or for those where the first function accounts for most of the variance, these tests can largely be interpreted as the singular contribution of the *p*th dependent variable.

## Appendix C Permutation details

The permutation approach implemented in the MRM software is based on the randomise algorithm published in Winkler et al. (2014), and on the multivariate approach detailed in Zeng et al. (2011). This approach allows for permutations in the presence of nuisance covariates. Firstly, we simplify the contrast **ABC**^'^ = 0 by re-expressing the test as **AB**^⁎^ = 0 in the model **Y**^⁎^ = **XB**^⁎^ + **E**^⁎^, where **Y**^⁎^ = **YC**^'^, **B**^⁎^ = **BC**^'^, and **E**^⁎^ = **EC**^'^ (Zeng et al., 2011). Although this may appear to simplify the model to univariate form, the number of columns in **Y**^⁎^ will be dependent on the rank of **C**. This is easiest to see when **C** = **I**_*t*_ as the model will not change. Once the model has been re-expressed, we use the model partition scheme from Ridgway (Ridgway, 2009; described in Winkler et al., 2014) to allow for permutation in the presence of nuisance covariates. Here we partition the model as **Y**^⁎^ = **MK** + **ZG** + **E**^⁎^ where **M** = **XA**^+^, **Z** = **X** - **XA**^'^(**A**^+^)', and superscript + denotes the Moore–Penrose pseudoinverse (Lay, 2012). Orthogonalising the effects of interest with respect to the nuisance partition is achieved by replacing **Z** with the first *m* columns of the left-hand matrix from the singular value decomposition (SVD) of **Z** (denoted **Z**_*s*_), where *m* = rank(**X**) - rank(**A**). Orthogonalisation of **M** is then achieved using **M**^⁎^ = **R**_*z*_**M** where **R**_*z*_ = **I** - **Z**_*s*_**Z**_*s*_^+^, the residual forming matrix due to the nuisance partition **Z**_*s*_. The hypothesis of interest, as originally coded in **A**, can now be tested by simply assessing whether **K** = 0. This can also be expressed in terms of testing the first *q* rows of parameters in the combined model $Y^{*}=\left[\begin{array}{cc} M^{*} & Z_{s} \end{array}\right]\theta +E^{*}$, where *q* = rank(**A**).

In terms of the steps involved, for each voxel we

1. Calculate the reduced model **Y**^⁎^ = **XB**^⁎^ + **E**^⁎^.
2. Partition the model into **Y**^⁎^ = **M**^⁎^**K** + **Z**_*s*_**G** + **E**^⁎^ as indicated above.
3. Estimate the reference test statistic using the estimated parameters $\hat{\theta }=V^{+}{\hat{\epsilon }}_{z}$ and residuals ${\hat{E}}^{*}=\left(I-VV^{+}\right){\hat{\epsilon }}_{z}$ from the combined model, where $V=\left[\begin{array}{cc} M^{*} & Z_{s} \end{array}\right]$, and ${\hat{\epsilon }}_{z}=R_{z}Y^{*}$, the residuals due to nuisance effects alone.
4. Randomly generate a permutation matrix **P** encoding row shuffles and sign-flips.
5. Estimate the test statistic using the parameters ${\hat{\theta }}_{p}=V^{+}P{\hat{\epsilon }}_{z}$ and residuals ${\hat{\epsilon }}_{p}=\left(I-VV^{+}\right)P{\hat{\epsilon }}_{z}$ of the permuted model.
6. Repeat steps 4–5 a sufficient number of times to build up an approximate null distribution of the test statistic of interest.

Using the sequence above we can calculate uncorrected *p*-values by counting the number of times at each voxel the permuted test statistic for that voxel exceeds the reference test statistic for that voxel. Dividing this by the number of permutations provides an estimated *p*-value for each voxel. These values could either be left uncorrected, or we can calculate *q*-values to provide standard FDR correction (Storey, 2002). For FWE correction, we simply count the number of times at each voxel the *largest* permutated test statistic in the image exceeds the reference test statistic for that voxel. Here we are building a distribution of the largest test statistic in an image, and the correction therefore equates to finding the probability under the null of a test statistic as large, or larger, than the reference *anywhere* in the image. Similarly, for cluster-based inference, on every reshuffle, we threshold the image using a parametric *p*-value to form clusters and save the size of the largest cluster. The corrected *p*-values for the clusters in the original image are then calculated based on the number of times across reshuffles the maximum cluster exceeded their reference size.

An important concept in permutation-based inference is *exchangeability*. Formally, exchangeability refers to the assumption that, for a particular set of variables, their joint probability distribution does not alter under rearrangement. For univariate approaches to dependent data exchangeability can prove problematic, requiring the specification of exchangeable blocks of data that can be shuffled as a whole. For the multivariate GLM, this issue is less problematic as we can simply shuffle the rows of the outcome matrix **Y**, or more generally **E**, but not the columns. This scheme is then identical to permutation of exchangeable blocks, given by the rows of **Y** or **E**, with sign-flipping performed as a whole within each block (Winkler et al., 2014). This can easily be seen by considering that for an *n* × *n* permutation matrix **P,** the multivariate permutations of the residuals **E,** given by **E**^⁎^ = **PE,** and the within-block permutations of the equivalent univariate residuals **ϵ** = Vec(**E**), given by **ϵ**^⁎^ = (**P** ⨂ **I**_*t*_)***ϵ,*** produce identical shuffles. Here, the latter is simply the univariate expression of the former so that **ϵ**^⁎^ = Vec(**E**^⁎^). We therefore assume *exchangeable*, *independent*, and *symmetric errors* for each row of data in **Y**. The assumption of symmetric errors can be relaxed by switching off sign-flipping. Although suitable for MANOVA contrasts, certain repeated-measures contrasts will no longer be permuted correctly given that any contrast that compares the estimated parameters for the columns of **Y** will not change under row swapping when sign-flipping is not performed. This is similar in spirit to the restrictions imposed on a one-sample *t*-test under univariate permutations.

## Appendix D Scanning parameters

In this appendix, we briefly detail the scanning parameters used when collecting the example datasets. All scans were collected using a Philips Achieva 3T at the Salford Royal NHS Foundation Trust. Data were acquired using a T2*-weighted EPI sequence with a TR of 2500 ms and a TE of 30 ms (matrix size 80 × 80, FOV 240 mm, flip angle 90°). Each volume consisted of 45 contiguous axial slices with voxel size of 3 × 3 × 3 mm. 290 volumes were collected in total. A T1-weighted structural image was also acquired (128 coronal slices, voxel size 1 × 1 × 1 mm, matrix size 256 × 164, FOV 256 mm, flip angle 8°).

## Appendix E Discrepancies between SPM 12 and GLM FLEX

As indicated in the text, a number of discrepancies between the test statistics given by SPM 12 and the test statistics given by GLM FLEX have been noted, despite the fact that both software packages implement the same repeated-measures ANOVA models. Of particular interest was the discrepancy between *F*_SPM_ = 41.35 and *F*_GLM_ = 45.84, seen at 0 20 47 in the main effect of the repeated measurement conditions. In this appendix, we outline the factors that appear to be influencing this difference. Largely, we believe that this not due to differences in numeric precision, rather it is due to the way the SPM non-sphericity correction is used in the different packages. Given that in the non-sphericity correction procedure the final parameters are estimated using the pre-whitened data and design, ${\hat{\beta }}_{W}={\left(\left[\mathrm{WX}\right]'\mathrm{WX}\right)}^{+}\left(\mathrm{WX}\right)'\mathrm{WY}$, the form that the whitening matrix **W** takes will directly influence the values of the estimates. Because, for the current example, we assumed covariance homogeneity, we can express **W** = **I**_*n*_ ⨂** K**, where *n* is the number of subjects. In the current example, the SPM estimate of **K** was

$$K_{\mathrm{SPM}}=\left(\begin{array}{ccccc} 1.0053 & 0.1379 & –0.1217 & –0.0211 & 0.0741\\ & 1.0058 & –0.0228 & –0.0686 & –0.0020\\ & & 1.0707 & –0.0015 & 0.0170\\ & & & 1.0755 & –0.0548\\ & & & & 0.9954 \end{array}\right)$$

whereas the **K** estimated by GLM FLEX is given by

$$K_{\mathrm{GLM}}=\left(\begin{array}{ccccc} 0.9538 & 0.1079 & –0.0999 & 0.0011 & 0.0314\\ & 0.9713 & –0.0113 & –0.0692 & –0.0101\\ & & 1.0661 & 0.0376 & 0.0113\\ & & & 1.1001 & –0.0613\\ & & & & 1.0369 \end{array}\right)$$

In terms of the subsequent influence on the parameter estimates in this voxel, the estimated values of interest (the factors and their interactions) only differed between SPM and GLM FLEX around the eighth decimal of the estimate. Differences were, however, seen in the estimates for each subject parameter, with a maximum discrepancy of around 0.0421. Because the residuals depend on **W**, as $\hat{\epsilon }=\mathrm{WY}-\mathrm{WX}{\hat{\beta }}_{W}$ , the estimate of the within-subject variance will also change by virtue of the differences in **W** and the differences in ${\hat{\beta }}_{W}$. In this voxel, the within-subject variance estimated by SPM was ${\hat{\sigma^{2}}}_{\mathrm{SPM}}=0.0821$, and in GLM FLEX was ${\hat{\sigma^{2}}}_{\mathrm{GLM}}=0.0837$.

In terms of the discrepancies in the *F*-test, we first review the standard formula for a univariate *F*-test within the pre-whitening framework

$$F=\frac{{\left(L{\hat{\beta }}_{W}\right)}^{'}{\left(L{\left[{\left(WX\right)}^{'}\mathrm{WX}\right]}^{+}L\right)}^{-1}\left(L{\hat{\beta }}_{W}\right)}{r\;\hat{\sigma^{2}}}$$

Already we can see how differences in the estimation of **W** will enter into this calculation. The numerator of this statistic given by SPM is 13.5753, and by GLM FLEX is 15.3504. Dividing these values by the estimated model variances, scaled by *r* = rank(**L**), provides the different test statistics highlighted earlier (when calculated at double precision). From this, we can see that the discrepancies between the SPM and GLM FLEX approaches are largely due to their respective implementations of the SPM non-sphericity correction, and the subsequent pre-whitening.

From our investigations, the difference in implementation between GLM FLEX and SPM centres on which voxels are selected to enter the pooled covariance estimation. This is evidenced by the fact that in the current example, GLM FLEX pools 45,128 voxels, whereas SPM pools only 2623. This is due to GLM FLEX using **I**_*k*_ as the contrast for selecting voxels to pool,^4^ where *k* is the number of columns in **X**. When SPM is forced to use this contrast by setting SPM.xVi.Fcontrast = eye(size(SPM.xX.X,2))′ the same number of voxels as GLM FLEX are used in the estimation procedure, the degrees of freedom for the initial *F*-test agrees with GLM FLEX, and **W** is nearly identical across the software. Though **I**_*k*_ may seem like a reasonable contrast to use, in the overparameterised framework, it is not an estimable function of the model (Searle, 1987). It is therefore debatable how sensible a test this is for selecting voxels to use in the pooled covariance estimation. By comparison, the ‘effects of interest’ contrast generated by SPM *is* an estimable function of the model and would therefore seem the better choice. These results also highlight one of the major problems with the pooling approach as the number of voxels entering the estimation directly influences the estimated whitening matrix and thus the estimated parameters of the model. In the current example, this difference leads to 781 more voxels surviving thresholding in GLM FLEX compared with SPM.

## Footnotes

1 We use the term multimodal generically to cover both multiple sequences from the same imaging modality (e.g. fMRI, ASL, DTI) as well as the different imaging modalities themselves (e.g. MR, PET).

2 This is in fact only true of the older GLM FLEX 2. The latest incarnation of GLM FLEX (known as GLM FLEX FAST) does not use the non-sphericity correction, and thus suffers from the same issues as FSL FEAT. Here we only focus on the older GLM FLEX 2.

3 It is also possible to fit these partitioned error-term ANOVA models in SPM using more basic group-level models by applying the Kronecker product rules outlined in Penny & Henson (2007).
