# Corrigendum to “Multivariate and repeated measures (MRM): A new toolbox for dependent and multimodal group-level neuroimaging data” [NeuroImage 132 (2016) 373–389]

**DOI:** 10.1016/j.neuroimage.2016.03.057

**Published:** 2016-08-15

**Authors:** Martyn McFarquhar, Shane McKie, Richard Emsley, John Suckling, Rebecca Elliott, Stephen Williams

**Affiliations:** a Neuroscience & Psychiatry Unit, Stopford Building, The University of Manchester, Oxford Road, Manchester M13 9PL, UK; b Centre for Biostatistics, Jean McFarlane Building, The University of Manchester, Oxford Road, Manchester M13 9PL, UK; c Brain Mapping Unit, Herchel Smith Building for Brain and Mind Sciences, University of Cambridge, Robinson Way, Cambridge CB2 0SZ, UK; d Imaging Sciences, Stopford Building, The University of Manchester, Oxford Road, Manchester M13 9PL, UK

The authors regret that a number of typographical errors made it into the final figure captions.

1. On page 380, the caption for Fig. 5 should read

Examples of the results found for 3 different contrasts from the 4 software packages. (a) The main effect of age, (b) the main effect of the repeated measurement conditions, (c) the age by repeated measurement condition interaction. All contrasts are thresholded at *p* < 0.01 to best visualise the differences between the software packages. The values underneath each image indicate the number of voxels remaining after thresholding. All statistics are presented as *F*-values. *Note* these results come from the *restricted* model comparisons where covariance homogeneity is assumed.

2. On page 381, in the caption for Fig. 6, the sentence beginning “These values can…” should read

These values can then be used to calculate $\hat{\beta }$ and $\hat{\mathrm{Var}}\left(\hat{\beta }\right)$ using a generalised least squares scheme, providing identical results to the pre-whitening approach (see Faraway, 2005, p. 89; Poldrack et al., 2011, p. 196).

3. On page 385, the caption for Fig. 10 should read

Comparisons between the four different multivariate test statistics for a non-exact multivariate test (a) thresholded at *p* < 0.01 using *p*-value approximations and (b) thresholded at *p* < 0.01 using the *p*-values derived from 5000 permutation tests. Results are presented as *p*-values transformed using − log_10_. The numbers above each image indicate the number of voxels that survive thresholding.

In addition, the reference for Faraway (2005) was accidentally missed from the reference section, and is now given below

Faraway, J., 2005. Linear Models with R. CRC Press, London.

The authors would like to apologise for any inconvenience caused.

